# Development and pregnancy rates of *Camelus dromedarius*-cloned embryos derived from *in vivo*- and *in vitro*-matured oocytes

**DOI:** 10.5713/ab.21.0131

**Published:** 2021-06-24

**Authors:** Young-Bum Son, Yeon Ik Jeong, Yeon Woo Jeong, Per Olof Olsson, Mohammad Shamim Hossein, Lian Cai, Sun Kim, Eun Ji Choi, Kenichiro Sakaguchi, Alex Tinson, Kuhad Kuldip Singh, Singh Rajesh, Al Shamsi Noura, Woo Suk Hwang

**Affiliations:** 1 UAE Biotech Research Center, 30310 Al Wathba, Abu Dhabi, United Arab Emirates; 2 Hilli E.T. Cloning and Surgical Centre Presidential Camels and Camel Racing Affairs, 17292 Al-Ain, United Arab Emirates

**Keywords:** *Camelus dromedarius*, Embryo Development, *In vitro*-matured Oocytes, *In vivo*-Matured Oocytes, Pregnancy Rates, Somatic Cell Nuclear Transfer

## Abstract

**Objective:**

The present study evaluated the efficiency of embryo development and pregnancy of somatic cell nuclear transfer (SCNT) embryos using different source-matured oocytes in *Camelus dromedarius*.

**Methods:**

*Camelus dromedarius* embryos were produced by SCNT using *in vivo*- and *in vitro*- matured oocytes. *In vitro* embryo developmental capacity of reconstructed embryos was evaluated. To confirm the efficiency of pregnancy and live birth rates, a total of 72 blastocysts using *in vitro*- matured oocytes transferred into 45 surrogates and 95 blastocysts using *in vivo*- matured oocytes were transferred into 62 surrogates by transvaginal method.

**Results:**

The collected oocytes derived from ovum pick up showed higher maturation potential into metaphase II oocytes than oocytes from the slaughterhouse. The competence of cleavage, and blastocyst were also significantly higher in *in vivo*- matured oocytes than *in vitro*- matured oocytes. After embryo transfer, 11 pregnant and 10 live births were confirmed in *in vivo*- matured oocytes group, and 2 pregnant and 1 live birth were confirmed in *in vitro*- matured oocytes group. Furthermore, blastocysts produced by *in vivo*-matured oocytes resulted in significantly higher early pregnancy and live birth rates than *in vitro*-matured oocytes.

**Conclusion:**

In this study, SCNT embryos using *in vivo*- and *in vitro*-matured camel oocytes were successfully developed, and pregnancy was established in recipient camels. We also confirmed that *in vivo*-matured oocytes improved the development of embryos and the pregnancy capacity using the blastocyst embryo transfer method.

## INTRODUCTION

*Camelus dromedarius* (Camel) is a desert animal that supplies milk and meat and is also gaining attention in racing and beauty applications [1]. However, the maintenance of the population due to the low reproductive efficiency of camels has been highlighted as a problem. *In vitro* embryo production (IVP) by somatic cell nuclear transfer (SCNT) is a useful technique to generate various kinds of animals with outstanding factors [2,3]. Since the first cloned camel was produced using *in vivo*-matured oocytes by ovum pick-up (OPU), a few studies have reported camel cloning [4–6].

In camels, collection of good quality oocytes is difficult due to limited ovaries obtained from the slaughterhouse, and camels were usually aged when they were slaughtered [1,6]. Therefore, cumulus oocyte complexes (COCs) collected from the ovary do not undergo *in vivo* developmental process, and highly heterogeneous oocytes are obtained. One of the main problems was that few studies on camel IVM system have been reported and the efficiency is also insufficient. Additionally, these oocytes showed variable developmental capacity after maturation [7]. In contrast to these slaughterhouse oocytes, which required *in vitro* maturation (IVM), *in vivo*-matured oocytes obtained by OPU do not require the development of final preovulation and produced a higher quality of embryos [8]. In addition, *in vivo*-matured oocytes collected using the OPU method can be repeatedly recovered from the same live animals. Therefore, the ultrasound-guided OPU method combined with the IVP of embryos has been widely used. Several studies have been reported on oocytes derived from OPU and slaughtered ovaries [7,8].

Despite recent studies on camel cloning, its low efficiency has produced few offspring. The improper reprogramming of donor nuclei injected into the enucleated oocyte cytoplasm may result in aberrant early embryo development, implantation problems and stillbirth [9]. Various types of factors affect reprogramming, including donor cell type [5], activation of oocytes [6], and oocyte cytoplasm [6]. Among them, the quality of oocytes is an important factor to consider for improving embryo development and producing offspring [4,6, 10–13]. However, studies on embryo development and pregnancy rate according to oocyte sources are limited in camels. Several studies have reported that the quality of the oocyte cytoplasm is different depending on the source of oocyte [6–8,10]. The capacity of embryo development collected with OPU-derived, *in vivo*-matured oocytes showed increased cleavage and blastocyst formation [6,10,14,15]. These results clearly indicated that *in vivo*-matured oocytes collected by OPU had high potential compared with oocytes obtained from slaughtered nonstimulated camel ovaries. Some previous studies reported that no difference in pregnancy rate depending on the sources of the oocytes but they used only a small number of oocytes and surrogates [4,6]. Furthermore, several studies on the efficiency of pregnancy have reported that the *in vitro* matured oocytes showed decreased pregnancy rates in other mammalians, including sheep, mouse, and human [11–13]. Therefore, the present study used a large number of surrogates, slaughtered ovaries-, and OPU-derived oocytes to reconfirm the effect of the source of oocytes on pregnant efficiency.

Described above, the present study was designed to determine the effects of oocyte sources on maturation, embryonic development, and pregnancy rates in camels. We established skin fibroblast cell lines which was used as donor nuclei, and we used *in vivo* matured oocytes collected by OPU technique and *in vitro* matured oocytes collected from ovaries from slaughterhouse. We performed SCNT to investigate the embryonic developmental capacity to progress to cleavage and blastocyst stages. Finally, we evaluated the pregnancy rates from these reconstructed embryos using blastocyst embryo transfer.

## MATERIALS AND METHODS

### Chemicals and media

All chemicals were purchased from Sigma (St. Louis, MO, USA) unless otherwise specified.

### Care and use of animals

This study was conducted from November 2018 to January 2019 when the estrus of camels occurred. We selected female camels without abnormalities in the reproductive tract to use as oocyte donors and surrogates. In this study, a total of 134 camels (107 surrogates, 27 oocyte donors) aged 4 to 7 years and weighing 400 to 450 kg were used. They were fed appropriate nutrients and provided water *ad libitum* daily. All animal procedures were conducted following the animal study guidelines, which were approved by the ethics committee at the Management of Scientific Centers and Presidential Camels (Accession No: PC4.1.5). The animal guidelines comply with the ARRIVE guidelines and were performed under the U.K. Animals (Scientific Procedure) Act, 1986, and associated guidelines, EW Directive 2010/63/EU.

### Transvaginal ultrasound-guided ovum pick-up

The oocyte donors were injected with 5,000 IU of PMSG (Ceva, Libourne, France) and 500 μg of closprostenol (Jurox, Rutherford, Australia) to stimulate the ovary as previously described with minor modifications [16]. We checked for superovulation based on the follicle diameter reaching 10 to 20 mm using ultrasonic assessment in camels after 9 days. Then, we treated camels with 100 μg of gonadorelin acetate (Vetoquinol, Paris, France) 25 to 28 hours before OPU was performed. The oocyte donors were sedated with 100 mg of ketamine (Ilium, Glendenning, Australia) and xylazine (Ceva, France). The oocytes were collected by an Aloka ultrasound unit (Aloka, Tokyo, Japan) with a needle guide (Aloka, Japan). We used a 60 cm, 18-gauge lumen needle in the follicles, along with 15 mL capped tubes with 2 mL OPU solution (IVF Bioscience, Falmouth, UK) using a regulated vacuum pump. All oocytes and follicular fluid were moved to petri dishes to identify COCs using a microscope. All oocyte donors were used only once.

### Collection of oocytes from slaughtered ovaries

In the present study, a total of 142 ovaries were used. We collected slaughtered ovaries from the Al-Ain Municipal slaughterhouse and transported them to the laboratory in 0.9% saline solution at 37°C. After that, COCs were recovered from ovaries as previously described with minor modifications [6]. In brief, we washed ovaries with 0.9% saline solution and aspirated follicles using an 18-gauge needle attached to a 10-mL disposable syringe. The collected COCs were evaluated as grade A and B according to the homogeneity of the cytoplasm and their enclosure by at least three layers of compact cumulus cells. They were washed three times in Dulbecco’s phosphate-buffered saline (DPBS; Welgene, Gyeongsan, Korea) supplemented with 5 mg/mL bovine serum albumin (BSA; Thermo Fisher Scientific, Waltham, MA, USA) and 1% (v/v) antibiotic-antimycotic (Thermo Fisher Scientific, USA). For IVM, these COCs were cultured at 38°C with 5% CO_2_ in a humidified atmosphere for 42 hours with commercially available BO IVM media (IVF Bioscience, UK).

### Establishment of donor cells

Ear skin samples were obtained from three female camels, and fibroblast cells were isolated as previously reported with minor modifications [17]. The biopsied tissue was washed with DPBS supplemented with 1% (v/v) antibiotic-antimycotic and minced into small pieces with scissors. The tissues were digested in Dulbecco’s modified Eagle’s medium (DMEM; Thermo Fisher Scientific, USA) supplemented with 0.1% (w/v) collagenase type IV (Thermo Fisher Scientific, USA) at 38°C in a humidified atmosphere of 5% CO_2_ for 2 hours. After that, the cells were washed with DPBS and filtered through 100- and 40-μm nylon strainers (Falcon, Franklin, NJ, USA). The cells were cultured in DMEM supplemented with 10% (v/v) fetal bovine serum (FBS) (Thermo Fisher Scientific, USA), 1% (v/v) nonessential amino acids (Thermo Fisher Scientific, USA), 1% (v/v) antibiotic-antimycotic (Thermo Fisher Scientific, USA) and 0.1% (v/v) β-mercaptoethanol (Thermo Fisher Scientific, USA) at 38°C in a humidified atmosphere of 5% CO_2_. The culture media was changed every two days until 80% confluence, and the cells were passaged with 0.25% trypsin ethylenediaminetetraacetic acid solution and frozen in DMEM supplemented with 20% (v/v) FBS and 10% (v/v) dimethyl sulfoxide.

### Somatic cell nuclear transfer

**Figure 1:**
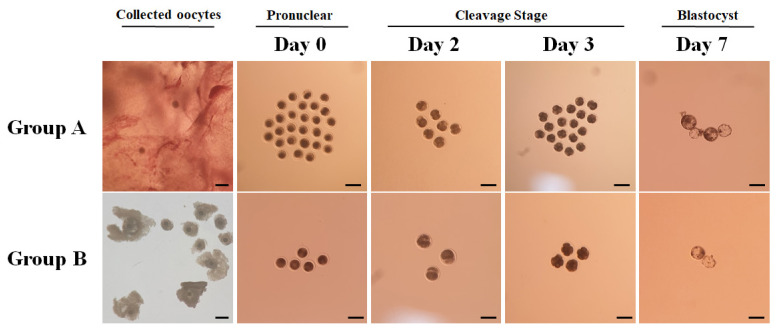
Embryo development after somatic cell nuclear transfer (SCNT). Oocytes were obtained by ovum pick up (OPU) (Group A), and from slaughtered camel ovaries (Group B). Embryo morphology was observed by a phase-contrast microscope on days 0, 2, 3, and 7. Scale bar = 300 μm.

The SCNT was performed by methods previously reported with minor modifications [18]. Briefly, we denuded cumulus cells from oocytes by gentle pipetting with 0.1% hyaluronidase. After denuding, MII phase oocytes were stained with 5 μg/mL bisbenzimide for 3 min. The stained oocytes were enucleated by aspirating polar body and metaphase II chromosome-containing ooplasm, and a single fibroblast was microinjected into the perivitelline space of the enucleated oocytes. The donor cell-oocyte couplets were fused in fusion media supplemented with 0.26 M mannitol, 0.1 mM MgSO_4_, 0.5 mM hydroxyethyl piperazine ethane sulfonicacid, and 0.05% (w/v) BSA with two DC pulses of 1.8 kV/cm for 15 μsec using a BTX Electro Cell Manipulator (BTX Inc., San Diego, CA, USA). After that, we treated the reconstructed embryos with 5 μM ionomycin for 3 min and with 2.0 mM 6-dimethylaminopurine (6-DMAP) in BO-IVC (IVF Bioscience, UK) in a humidified incubator with 5% CO_2_ at 39°C for 4 hours. Following activation, the embryos were cultured in an oil-covered BO-IVC droplet of 6 to 8 at 38°C in a humidified atmosphere with 5% CO_2_ and 5% O_2_. We checked early stage embryo development up to 8-cell at day 2 and day 3 and blastocyst formation was confirmed at day 7 (Figure 1).

### Embryo transfer and pregnancy diagnosis

The recipients were prepared in the same way as the oocyte donor for OPU described above. We sedated camels with 100 mg of xylazine (Ceva, France). The preparation of the blastocyst stage embryo transfer was also similar to that of OPU donors. One to two cloned day 7 blastocysts were transferred with transfer media to either horn of the uterus.

To confirm pregnancy, we carried out a tail response test on the surrogates 10 days later and analyzed the serum progesterone concentration 16 days after embryo transfer using a chemiluminescence immunoassay (Roche, Basel, Switzerland). After that, real-time ultrasonography was performed with the camel in a standing position every 30 days.

### Statistical analysis

Statistical analyses were performed by independent T-test of variance using SPSS version 23 (IBM) for between-group comparisons. Data are presented as the mean±standard error, and p<0.05 was considered significant.

## RESULTS

### Effect of the source of oocytes on oocyte maturation and the development of somatic cell nuclear transfer embryos

**Figure 2:**
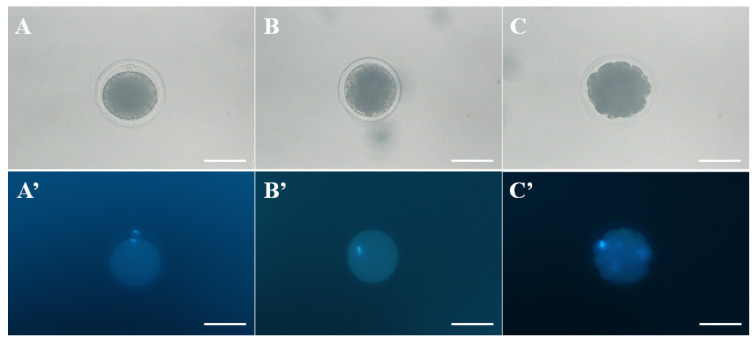
Morphology of normal and abnormal matured oocyte was observed by phase contrast- and fluorescence microscope. A and A′. The morphologies of a normal metaphase II (MII) oocyte, B and B′. Metaphase I (MI) oocyte, and C and C′. abnormal matured oocyte (degradation of cytoplasm). Oocytes were stained with Hoechst 33342 to confirm the DNA. Scale bar = 100 μm.

To clone *Camelus dromedarius*, a total of 862 oocytes were collected from slaughtered nonstimulated camel ovaries, and 347 oocytes were collected by OPU. The IVM efficiency of oocytes derived from the slaughterhouse was compared with the *in vivo* maturation efficiency of oocytes derived from OPU (Table 1; Figure 2). We confirmed the significantly (p<0.05) higher maturation potential in the collected oocytes derived from OPU compared with oocytes from the slaughterhouse. The results of fused oocytes and cleavage and blastocyst embryos are presented in Table 2. Among the SCNT embryos the cleavage rates of SCNT embryos derived from *in vivo*-matured oocytes were significantly (p<0.05) higher than those from *in vitro*-matured oocytes. Furthermore, the rate of blastocyst development was significantly (p<0.05) higher in the *in vivo*-matured oocyte group than in the *in vitro*-matured oocyte group.

### Pregnancy of cloned camels and identification

Camel cloned embryos were constructed from both kinds of oocytes, and pregnancy was confirmed through ultrasonography 30, 60, and 90 days after embryo transfer. We performed embryo transfer using day 7 blastocyst stage embryos. A total of 72 blastocysts using *in vitro*-matured oocytes were transferred to 45 surrogates, and 95 blastocysts using *in vivo*-matured oocytes were transferred to 62 surrogates (Table 3). Among the 45 surrogates, 2 pregnancies were detected, and 1 reached live birth. Among the 62 surrogates, 11 pregnancies were detected, and 10 reached live birth (Table 3). The efficiencies of pregnancy and live birth were significantly increased in the *in vivo*-matured oocyte group compared with the *in vitro*-matured group (Table 3).

## DISCUSSION

In the present study, we evaluated the development of SCNT embryos and term after transfer using *in vivo*- and *in vitro*-matured camel oocytes. Several studies have reported the influence of oocyte sources from various mammals on embryo development [1,6,10,14,15]. The development of SCNT embryos must be investigated in terms of oocyte maturation, embryo cleavage, and blastocyst formation capacity. In bovines, an increased embryo production rate was identified after IVF using *in vivo*-matured oocytes compared with *in vitro*-matured oocytes [10]. Moreover, increased embryo production rates were identified after IVF using *in vivo*-matured oocytes compared to *in vitro*-matured oocytes in mice and monkeys [14,15]. The decreased rates of cleavage and blastocyst stage with *in vitro*-matured oocytes might have been influenced by oocyte cytoplasm quality and factors caused by the time wasted during ovary acquisition and transportation [19]. Furthermore, the collection of ovaries from an unsanitary environment in a slaughterhouse, the status of heterogeneous oocytes, and difficulty maintaining genetic merit are all problematic factors [10]. The results of the present study were similar. We evaluated the cleavage and blastocyst stage formation capacities and determined that *in vivo*-matured oocytes had higher efficiency compared with *in vitro*-matured oocytes.

Other evidence has suggested that defects in camel oocyte IVM could affect oocyte quality and embryo development [4]. The capacities for embryo development and pregnancy were different between *in vitro*-matured oocytes and *in vivo*-matured oocytes because of their surrounding environment [20]. Additionally, oocyte development and maternal transcript synthesis occurred simultaneously [20,21]. It has been reported that *in vitro*-matured bovine oocytes showed decreased cell cycle regulation, oocyte maturation and oxidative phosphorylation-related gene expression with increased apoptosis-related gene expression compared with *in vivo*-matured oocytes [20,21]. These results indicated that it is essential to choose an efficient oocyte maturation method to obtain stable results in the production of live births using SCNT. However, the oocyte maturation rate of camels was lower than that of other species after IVM, and studies were insufficient [22–24]. According to recent studies, camels are generally slaughtered when they are too old or too young before their maturity; therefore, they do not undergo normal reproduction cycles and have been reported to have reduced embryo development capacities [1,6]. Therefore, we hypothesized that *in vivo*-matured oocytes would be more efficient in SCNT. Our results supported this hypothesis. The present study revealed a greater efficiency in obtaining matured oocytes from *in vivo*- compared to *in vitro*- sources (Table 1). Indeed, we confirmed the enhanced capacities for cleavage and blastocyst stage formation of *in vivo*-matured oocytes. This evidence might be one approach to the efficient acquisition of metaphase II oocytes for SCNT to obtain *in vivo*-matured oocytes.

The evaluation of pregnancy and live birth rates should be performed in SCNT embryo transfer. Some earlier studies reported that the source of oocytes in camels influenced embryo development, but no effect was observed on pregnancy rate [4,6]. These reports remain unclear due to limited oocyte, blastocyst, and surrogate numbers evaluated between OPU-derived and slaughterhouse oocytes [4,6]. Additionally, low comparative numbers of *in vivo*- to *in vitro*- embryo transfer render these comparisons at best incomplete, e.g. Moulavi et al [4] reported results from 5 embryo transfers in an *in vivo* group with 40 in their *in vitro* group, differences of more than eight times between groups. Wani et al [6] similarly reported data from groups that varied more than three times. Oocyte maturation rates are additionally reported in the present study, information absent in the aforementioned previous reports, providing a more complete understanding of the capacity and limitation of oocyte sources.

Several studies have evaluated such similar strategies using different maturation system of oocytes with various kinds of mammals, including sheep, mouse, and human [11–13]. In the case of sheep, SCNT embryos were transferred to surrogates, and an *in vivo*-matured oocyte group was determined to have high pregnancy and full-term development rates [13]. When human embryos transferred to the recipient, conventional IVF/ICSI using *in vivo*-matured oocytes showed higher pregnancy capacity than *in vitro*-matured oocytes [11]. Furthermore, oocytes after IVM showed high rate of pregnancy loss [25]. Several studies also reported to prevent a decrease in pregnancy rate according to the poor quality embryos after IVM, three or more embryos were transfer into surrogates [26,27]. One of the reasons was that chromosome abnormalities and incorrect spindle assembly affected the implantation of embryos [13,14]. Therefore, we performed embryo transfer using blastocyst stage embryos, assuming that in vivo-matured oocytes had enhanced potency in successfully establishing pregnancy. To confirm this hypothesis, the present study evaluated the effects of in vivo- and *in vitro*- matured oocytes on oocyte maturation capacity and pregnancy rates using a large number of different sources of oocytes and surrogates. We produced 11 full-term camels using SCNT embryos and confirmed that the in vivo-matured oocyte group showed higher pregnancy and live birth efficiencies using the blastocyst embryo transfer method (Table 3). This study did not elucidate the mechanisms of these results for oocytes and embryos. However, considering the low efficiency of the IVM system in camels, in vivo-matured oocytes were determined to be the most proper source for camel SCNT.

## Figures and Tables

**Table 1:** Effects of *in vivo*- and *in vitro*-matured oocytes on oocyte maturation capacity

Source of oocytes	Oocyte maturation			

	No. of oocytes			

	Collected oocytes	MII (%)^1)^	Immature (%)^2)^	Abnormal
*In vitro* matured oocytes	862	517 (60.1±1.0)^a^	319 (37.2±1.0)^a^	25 (2.62±0.4)
*In vivo* matured oocytes	347	309 (89.3±2.2)^b^	29 (8.6±2.1)^b^	9 (2.1±0.5)

1) MII, metaphase II oocytes.

2) Immature, germinal vesicle, germinal vesicle breakdown, and metaphase I oocytes.

a,b Different superscript letters represent significance (p<0.05).

**Table 2:** Effects of *in vivo*- and *in vitro*-matured oocytes on the embryo development capacity of somatic cell nuclear transfer in camels

Source of oocytes	Nuclear transfer			

	No. of oocytes			

	Reconstructed oocytes	Fused (%)^1)^	Cleaved (%)^2)^	Blastocyst (%)^3)^
*In vitro* matured oocytes	517	362 (71.2±1.7)	217 (59.7±3.2)^a^	73 (20.92±2.1)^a^
*In vivo* matured oocytes	309	223 (74.6±1.9)	183 (75.3±3.5)^b^	101 (45.7±2.3)^b^

1) The fusion rate was calculated by the number of reconstructed oocytes.

2), 3) The cleavage and blastocyst rates were calculated by the number of fused oocytes.

a,b Different superscript letters represent significance (p<0.05).

**Table 3:** Pregnancy rates from somatic cell nuclear transfer embryos using *in vivo*- and *in vitro*-matured oocytes on blastocyst stage embryo transfer to camels

Items	Source of oocytes		

	*In vitro*-matured oocytes	*In vivo*-matured oocytes	p-value
No. of transferred embryos	72	95	-
No. of surrogates	45	62	-
Pregnancy rate (D30)	2 (4.4%)	11 (17.7%)	0.034
Pregnancy rate (D60)	1 (2.2%)	10 (16.1%)	0.017
Pregnancy rate (D90)	1 (2.2%)	10 (16.1%)	0.017
Pregnancy rate (Live birth)	1 (2.2%)	10 (16.1%)	0.017

The rates of pregnancy were based on the number of surrogates.

Abortion accrued between 30 and 60 days.
